# “Any fool can make a rule and any fool will mind it”

**DOI:** 10.1186/s12916-016-0562-1

**Published:** 2016-02-10

**Authors:** Athol U. Wells

**Affiliations:** Interstitial Lung Diseases Unit, Royal Brompton Hospital, Sydney Street, London, SW3 6NP UK

**Keywords:** Diagnosis, Idiopathic pulmonary fibrosis, Guidelines, Clinical reasoning

## Abstract

In principle, accurate guideline recommendations should lead to optimal management based on a secure diagnosis. However, current IPF diagnostic guidelines do not meet the needs of a major sub-group (possibly the majority) of patients with idiopathic pulmonary fibrosis (IPF). A great many IPF patients have HRCT appearances of “possible UIP”. A surgical biopsy is very often impracticable due to age, disease severity, co-morbidities or patient refusal. A guideline-based diagnosis cannot be made in these patients, although the diagnosis is often obvious. Inflexible diagnostic criteria, although essential for treatment trials, must necessarily be structured around an inflexible diagnostic algorithm. With this approach, non-standardised information (i.e. not available in all patients) must be omitted, including observed disease behaviour prior to and on treatment, findings on bronchoalveolar lavage, likelihoods in relation to age and a wealth of ancillary clinical information. However, when a diagnosis cannot be made using guideline criteria, a probable or highly probable “working diagnosis” of IPF can and should be made in most IPF patients by means of clinical reasoning, integrating all available non-standardised information.

## Background

Idiopathic pulmonary fibrosis (IPF), the most progressive and most prevalent of the idiopathic interstitial pneumonias (IIPs), requires a radically different management from the other IIPs due to its prognostic implications and the advent of IPF-specific therapies. With regards to their management, the remaining IIPs can largely be conceptualised as forms of immune dysregulation; however, this model has failed in IPF [1].

It might be supposed that the accurate diagnosis of IPF should depend upon the application of evidence-based diagnostic guidelines. Yet, in at least half of IPF patients, the diagnosis cannot be made using current American Thoracic Society/European Respiratory Society/Japanese Respiratory Society/Latin America Thoracic Association (ATS/ERS/JRS/ALAT) guideline criteria [2]. The ‘failsafe option’ of a surgical biopsy, recommended when the diagnosis cannot be made non-invasively, applies to perhaps 15 % of patients in this setting given the average age of onset, severity of disease at presentation, comorbidities and, importantly, the disinclination of patients to undergo a procedure with a mortality rate of 3–6 % [3, 4]. It is possible that cryobiopsy techniques will make a major difference [5], but only time will tell.

Currently, in roughly half of IPF patients, ATS/ERS/JRS/ALAT 2011 diagnostic criteria for IPF are not satisfied and the disease remains unclassifiable. In this setting, clinicians must speculate on the best course of action – whether to manage the disease according to a diagnosis of alternative disorders or to apply IPF-validated anti-fibrotic therapies. In a nutshell, the ATS/ERS/JRS/ALAT 2011 IPF diagnostic guidelines are ‘broken’.

How has it come to this? The pace of recent events is partly to blame. In 2009/2010, when the ATS/ERS/JRS/ALAT guidelines were formulated, there was no universally accepted therapy known to delay disease progression and it was generally agreed that best management consisted of participation in a novel treatment trial [2]. Rigorous diagnostic criteria are required in drug trials. However, and perhaps more importantly, a treatment option, in the form of triple therapy with low dose prednisolone, azathioprine, and N-acetyl cysteine [6], was available. This option could, until recently, reasonably be applied equally in IPF or in the realistic differential diagnoses in which immune dysregulation is thought to play a primary pathogenetic role. Thus, the ATS/ERS/JRS/ALAT 2011 guidelines provided appropriate diagnostic rigour for trial purposes and, at that time, it could be argued that patients disenfranchised by IPF diagnostic criteria were not disadvantaged for practical management purposes. Perhaps it was not possible in 2011 to do better than this. However, everything changed irrevocably when IPF-specific therapies were validated, and particularly when it became apparent, from the findings in the PANTHER study, that triple therapy should not be used in IPF [7]. For the first time, diagnosis became pivotal in clinical practice, as opposed to the confines of drug trials. PANTHER was the final clause in the destabilisation of the 2011 IPF diagnostic guidelines.

Nevertheless, recent events are only partly to blame. There is a second issue related to the decline and fall of ‘eminence-based medicine’. Fortunately, the age of the opinion hegemony – a handful of experts driving best medical practice based on their anecdotal views – is over. However, we have fallen into a new and equally damaging trap – the belief that logic and common sense must be discarded and only data formalised within an ‘evidence base’ can be taken into account. It may seem surprising that the Evidence-based Renaissance Group recently published a perspective entitled, Evidence-based Medicine, a Movement in Crisis [8]. However, by definition, the rigid application of an evidence base is founded on the assumption that ‘one size fits all’. Sometimes, this approach is essential – in the evaluation of expensive treatments which may have serious side effects, a rigorous question must be asked using stringent methods. However, in other contexts, an evidence-based tyranny can actually be harmful, as, for example, in the multidisciplinary diagnosis of IPF.

Whatever else is said about the ATS/ERS/JRS/ALAT 2011 IPF guidelines, it cannot be doubted that, for the first time, an evidence-based ethos was energetically applied to the diagnosis and management of IPF. However, it was telling that nothing useful was said about that great desideratum, multidisciplinary diagnosis. Certainly, the phrase ‘multidisciplinary diagnosis’ makes cameo appearances from time to time, but what is described in the document as multidisciplinary diagnosis is unrecognisable to most practising clinicians, radiologists and histopathologists. The casual reader, focusing on tables and key conclusions, might be excused for imagining that the process of multidisciplinary diagnosis consists of amalgamating a histological grade and a high-resolution computer tomography (HRCT) category, each reached in isolation, providing a definition of possible, probable or definite IPF. In this model, the clinician has no particular role, apart from the exclusion of a primary cause of pulmonary fibrosis. The problem, here, is that diagnosis for trial purposes, enshrined in the 2011 guideline, differs radically from enlightened multidisciplinary diagnosis.

In treatment trials, it is essential that diagnosis is rigorous and standardised in order to minimise diagnostic variation between treatment arms. In essence, this means the use of simplistic clinical exclusion criteria and HRCT data, and a requirement for a diagnostic surgical biopsy when HRCT appearances are inconclusive. Rigid diagnostic criteria require the exclusion of all information not available in all patients. Observed disease behaviour, on and off therapy, does not feature as a possible influence on the likelihood of IPF in the ATS/ERS/JRS/ALAT 2011 guidelines because, at enrolment into a trial protocol, observations of disease behaviour are not always available. Bronchoalveolar lavage is not a part of the diagnostic algorithm in all countries and therefore it was relegated to an undefined ‘weak negative’ role in the ATS/ERS/JRS/ALAT 2011 guidelines. This is relevant because, in clinical practice, hypersensitivity pneumonitis exposures are not, in reality, a simple ‘present/absent’ dichotomy. Often, there is real difficulty in assigning significance to exposure. The whole question of what constitutes occult connective tissue disease is too complex to summarise concisely, but frequently contributes to the warp and weft of multidisciplinary diagnosis.

For trial purposes, diagnosis must necessarily be ‘dumbed down’. Multidisciplinary diagnosis is a different matter altogether. Trial diagnostic criteria require the consideration only of data standardisable in all patients. By contrast, multidisciplinary diagnosis requires the consideration of all potentially relevant information in every individual patient. Trial diagnostic criteria confine expert judgement to the isolated views of the radiologist and histopathologist, with subtle clinical interpretation disallowed. Multidisciplinary diagnosis consists of debate and the integration and reconciliation of a huge amount of ancillary clinical information. It is sometimes supposed that the major value of multidisciplinary diagnosis consists simply of broadening expertise. However, it can also be argued that the true value of a multidisciplinary team lies in bringing together trained minds accustomed to civilised disagreement and the scrutiny of logic and common sense. Above all, the use of logic, common sense and review of all data (which differs widely in its completeness in every patient) cannot be validated using an evidence-based approach in which inflexible diagnostic criteria are required.

## Conclusions

With the use of a multidisciplinary approach, a diagnosis of IPF can often be made, based on very high probability, in patients who lie outside guideline criteria. Anti-fibrotic therapy in this large patient subgroup can be instituted with the same confidence as in ‘definite IPF’, as defined in the 2011 guidelines. In other cases, in which IPF is merely the most likely of several possible diagnoses, an eventual working diagnosis of IPF and the introduction of anti-fibrotic therapy can be justified by subsequent disease progression despite immunomodulatory therapy. In this area, medicine is as much an art as a science. The evidence-base apparatchik need not despair – the time will come for the multidisciplinary approach in IPF to be subjected to evidence-based evaluation. The accuracy of a flexible approach to diagnosis, based on the integration of clinical reasoning and morphologic (CT and pathology) pattern recognition, can be examined against subsequent natural history and treatment course, given that the value of making a diagnosis is in the provision of this information. However, the answer does not lie in recreating new rigid diagnostic criteria for IPF to the detriment of clinical reasoning. As Thoreau, father of the pragmatic philosophy movement once said, “*any fool can make a rule and any fool will mind it*”.
